# Pediatric Sigmoid Volvulus: A Report on Two Cases

**DOI:** 10.7759/cureus.28400

**Published:** 2022-08-25

**Authors:** Varsha Madhavnarayan Totadri, Rishwanth Vetri, Surabhi Sainath

**Affiliations:** 1 General Surgery, Stanley Medical College and Hospital, Chennai, IND

**Keywords:** coloanal anastomosis, idiopathic pediatric sigmoid volvulus, normoganglionic, sigmoidectomy, coffee bean sign, large bowel obstruction, paediatric intestinal obstruction, redundant sigmoid colon, sigmoid volvulus

## Abstract

Sigmoid volvulus is a rare cause of intestinal obstruction in the pediatric age group. Rotation of the redundant sigmoid colon about its narrow mesenteric base results in vascular compromise and large bowel obstruction. Predisposing factors for sigmoid volvulus are Hirschsprung’s disease, congenital anomalous fixation of the colon, and chronic constipation. Here, we report two cases of sigmoid volvulus in children with redundant sigmoid colon in the South Indian subcontinent. If it is not diagnosed in time, it may lead to serious complications such as gangrene, perforation, septic shock, and eventually death. Thus, the condition warrants prompt evaluation and treatment.

## Introduction

Sigmoid volvulus is an uncommon problem in children and adolescents; hence, it is rarely considered as a diagnosis in this group because it is a classic disease of the elderly [1]. Volvulus occurs when a redundant sigmoid loop rotates around its narrow-based, elongated mesentery, resulting in arterial and venous obstruction of the affected segment, and subsequent rapid distention of the closed loop [2]. Since the consequences can be severe, sigmoid volvulus should be included in the differential diagnosis of acute and recurrent episodes of abdominal pain or bowel obstruction in this population, particularly if colonic dilation is observed on radiographs. The most common symptoms are abdominal pain relieved by passage of stool or flatus, abdominal distention, and vomiting. Volvulus in the pediatric population commonly occurs in the small bowel associated with malrotation or internal hernia, whereas sigmoid volvulus is very rare [3]. There are only a few cases reported to date. Herein, we report two cases of acute sigmoid volvulus in 12-year-old boys, both presenting with intestinal obstruction wherein the diagnosis was strongly suggestive of sigmoid volvulus as per the abdominal radiographs.

## Case presentation

Case 1

**Figure 1:**
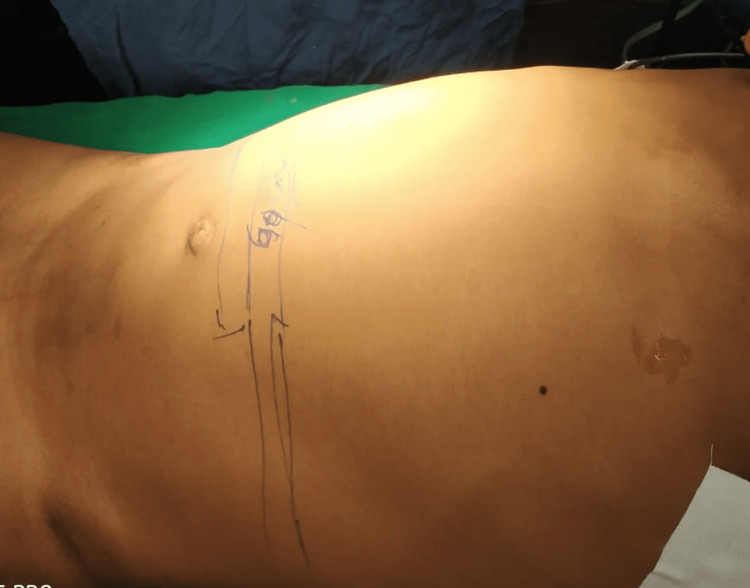
Clinical photo showing abdominal distension

A 12-year-old boy, Master X, with no previous disease/comorbidities, presented to the emergency department with complaints of abdominal pain and abdominal distension (Figure 1), obstipation, and multiple episodes of vomiting 20 minutes after consuming food for the past three days. The pain was colicky in nature, initially intermittent and thereafter becoming constant. He had no history of recurrent episodes of constipation or difficulty in the passage of stools. His birth and development history were noted to be normal, with no specific history of late passage of meconium or any operative or invasive procedures. Clinical examination revealed a moderately built and nourished child with Tanner stage 2, and upper abdominal distension. The child was noted to have tachycardia, tachypnoea, dyspnea, and signs of moderate dehydration. Abdominal examination revealed upper abdominal distension with umbilicus in the midline, guarding and absent bowel sounds with digital rectal examination revealing roomy rectum. Blood and biochemical parameters were within the normal limits.

**Figure 2:**
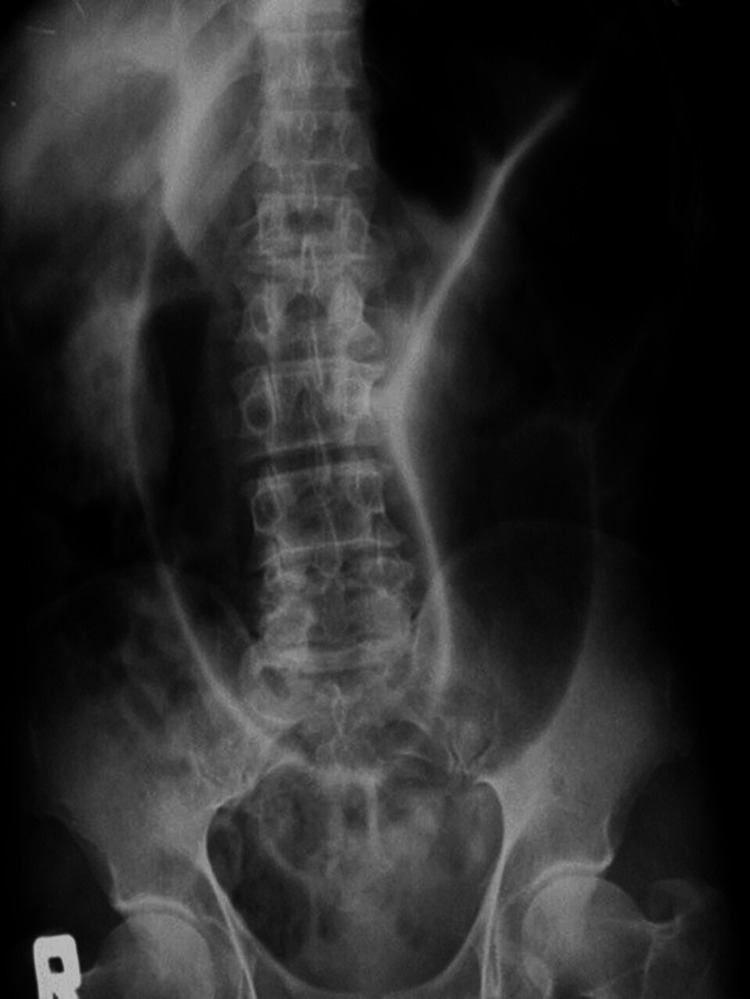
"Coffee Bean" sign on erect abdominal radiograph.

A plain radiograph (Figure 2) of the abdomen in standing posture revealed a massively dilated colonic loop with a classical “Coffee Bean” sign with an elevated left hemidiaphragm giving a strong suspicion of Sigmoid Volvulus. Computed tomography (CT) of the abdomen and pelvis reinforced the diagnosis-showing that the sigmoid along with its mesocolon, appeared swirled with proximal large bowel loop dilatation. Due to the unavailability of emergency endoscopy, endoscopic detorsion could not be attempted and as per the surgeon's decision, the child was thereafter taken up for emergency laparotomy.

**Figure 3:**
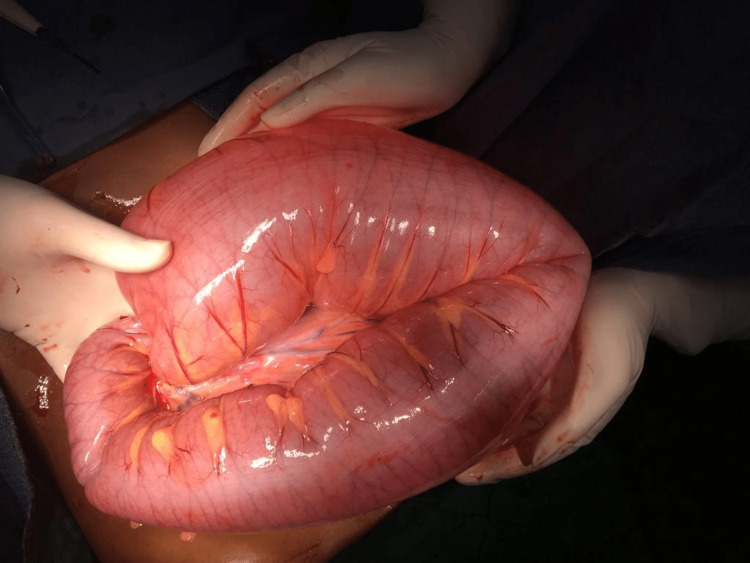
Massively dilated Sigmoid colon

Intra-operative findings revealed a Sigmoid Volvulus of 360° around its mesentery (Figure 3), and the affected segment showed dilatation and edema. The twisted part of the sigmoid colon appeared longer than expected in a pediatric age group, being the cause of the volvulus. No evidence of gangrene noted. Viability of bowel was doubtful, and as sigmoid colon with mesentery was longer than expected for paediatric age group, therefore, the child underwent sigmoidectomy with primary end-to-end anastomosis.

Post-operatively, vitals remained stable and abdominal distension reduced. Respiratory effort improved post-operatively with no further episodes of respiratory distress. Bowel sounds reappeared on postoperative day 2, and the child passed flatus on the same day, following which the child was started on enteral feeds (oral diet) with an initial liquid diet. The child began passing formed stools from postoperative day 3. The wound was healthy, and the remainder postoperative period was uneventful. The child was discharged on postoperative day 7. Postoperative histopathology report indicated no evidence of aganglionic segment.

Case 2

**Figure 4:**
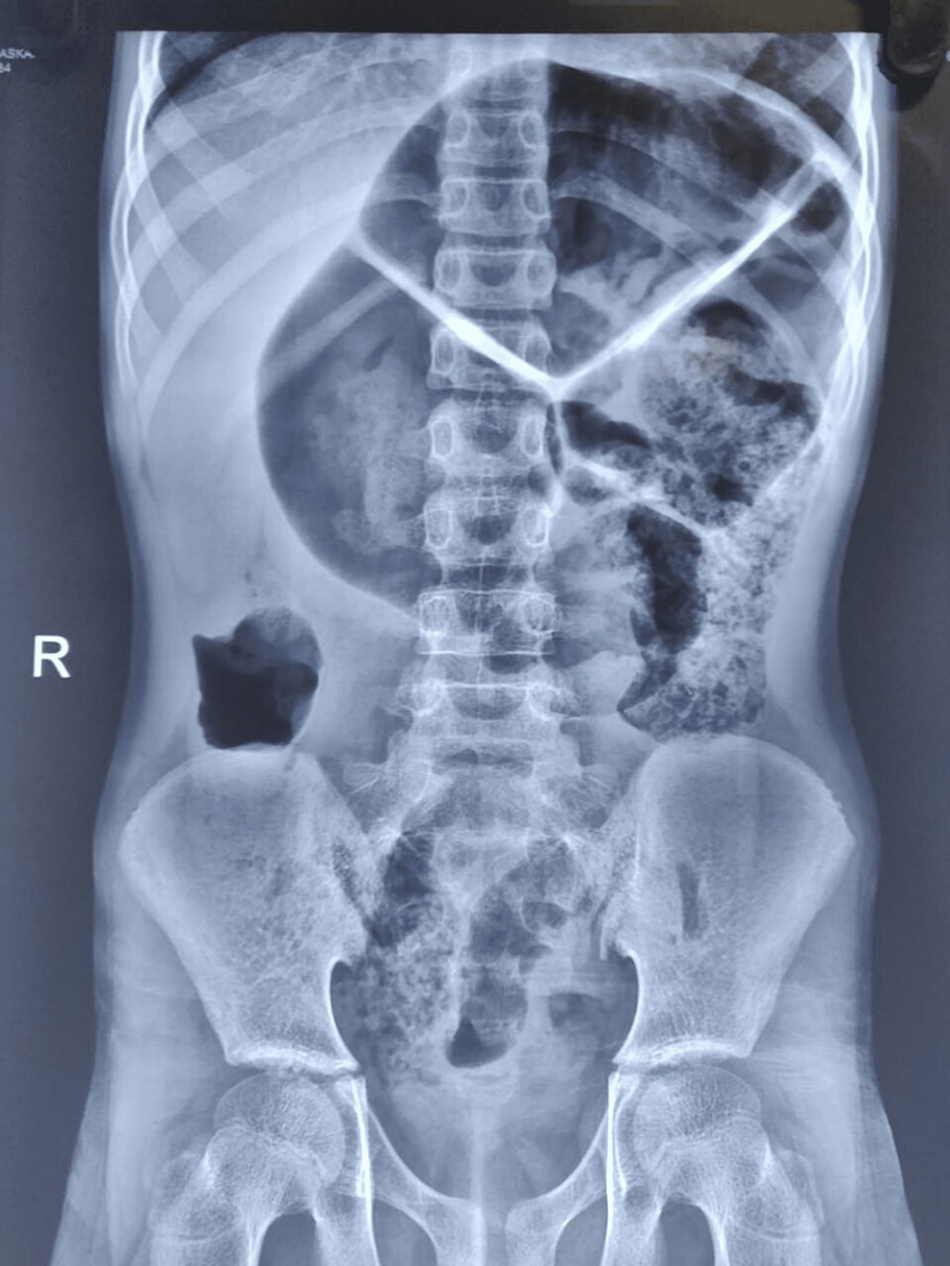
“Coffee bean sign” on X-ray abdomen erect

Another 12-year-old boy, Master Y, with no previous disease/comorbidities, presented to the emergency department with complaints of abdominal pain for 10 days. The pain was intermittent and colicky in nature with aggravation over the last two days. Furthermore, three episodes of vomiting, containing undigested food particles, non-blood or bile stained, occurring half an hour after the consumption of food and obstipation over the last two days were reported. He complained of gradual abdominal distension over the past two days. He had a history of a single episode of loose stools 10 days prior, normal color, non-blood stained/non-foul smelling, and no melena. He had no history of recurrent episodes of constipation. Birth and development history was normal with no history of delayed passage of meconium or stunted development. Clinical examination revealed a moderately built and nourished boy with Tanner staging 2 and upper abdominal distension. The child had tachycardia and moderate dehydration. Abdominal examination revealed upper abdominal distension with umbilicus in the midline, localized guarding over the epigastric region, diffusely tender left lumbar and hypochondrium, and sluggish bowel sounds; digital rectal examination revealed a roomy rectum. Blood and biochemical parameters were within normal limits. A plain radiograph of the abdomen in standing posture (Figure 4) was obtained and revealed dilated colonic loop with a classical “Coffee Bean” sign strongly indicative of Sigmoid Volvulus.

**Figure 5:**
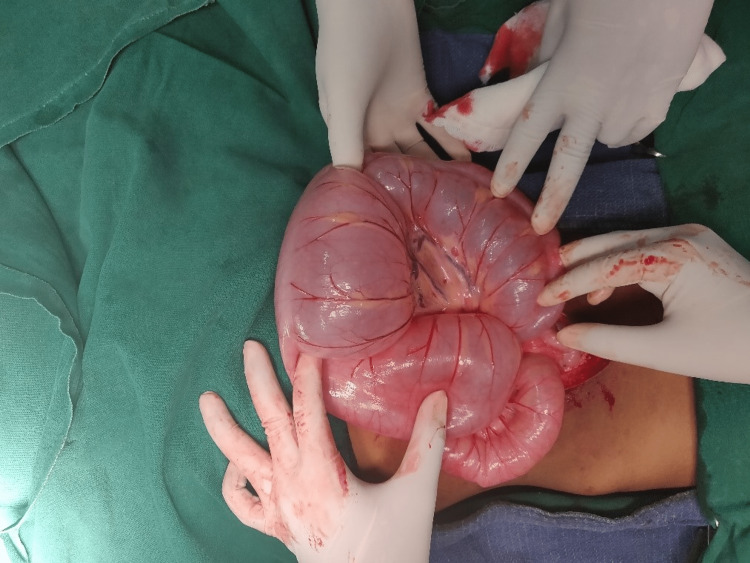
Sigmoid volvulus with dilated large bowel loops

After resuscitation, the patient was proceeded for emergency laparotomy due to the lack of an endoscopic intervention provision at the centre. Intra-operative findings revealed a Sigmoid volvulus of 540°, forming three loops of the sigmoid colon around its axis with massive dilatation and congestion but no evidence of gangrene (Figure 5). Detortion of the sigmoid volvulus was performed, and the sigmoid colon with mesentery was much longer than expected for the pediatric population (thereby demonstrating redundant colon with mesentery even if decompression with sigmoidopexy was considered). In view of the long redundant sigmoid colon with mesentery and no peristaltic movement, viability of the bowel remained doubtful. Hence sigmoidectomy with primary end-to-end anastomosis was proceeded with.

Post-operatively, vitals remained stable and abdominal distension reduced. Bowel sounds re-appeared on postoperative day 2. The child passed flatus from postoperative day 3 and passed stools on postoperative day 4. Enteral feeding was reinstated from postoperative day 5, and the child improved clinically. The postoperative period remained uneventful, and the patient recovered well. Post Operative Histopathology report showed no evidence of aganglionic segment.

## Discussion

Volvulus can develop at any segment of the large bowel, but it is commonly seen in the sigmoid colon due to the long mesentery and inherent anatomy. However, volvulus in the sigmoid colon in the pediatric age group is a rare entity [4]. Chronic constipation and neuropsychiatric disorders are common occurrences associated with sigmoid volvulus in the elderly. Moreover, a high fiber diet, motility disorders, and pregnancy have been noted to be concurrent with volvulus. In the pediatric age group, colonic volvulus has been associated with Hirschsprung's disease [5].

Only a few cases/case series of sigmoid volvulus in the pediatric population have been documented worldwide in literature. In a study conducted between 1941 to 2000, Salas et al. reported only 63 cases, with a median age of seven years and a male preponderance (male:female ratio, 3.5:1) [6].

Sigmoid volvulus occurs when a redundant loop of the sigmoid rotates around the elongated and narrow mesentery, causing venous and arterial obstruction of the involved segment and rapid distension of the closed-loop [4]. If not managed promptly, it can lead to dire consequences such as gangrene, perforation, and septic shock [3]. Obstruction of the lumen occurs when the torsion is more than 180°, and eventually, perfusion impairment occurs when the rotation of the sigmoid around its axis reaches 360°. Although sigmoid volvulus as an entity is common in India, Puneet et al. reported only six cases of sigmoid volvulus in children under the age of twenty over 10 years of study [7].

Predisposing factors in children have been studied over the years in the few cases documented in the literature, wherein Hirschsprung's disease has been a common association due to short aganglionic segment and dilated ganglionic segment with freely mobile mesentery [8,9]. Congenital anomalous fixation of the colon, myotonic dystrophy, and prune belly syndrome are the other causative factors.

The diagnosis of sigmoid volvulus requires a detailed history, clinical examination, and appropriate interpretation of plain abdominal radiographic films. The diagnostic findings include a "whirlpool" sign caused due to the dilated sigmoid colon around its mesocolon and vessels and "Bird's beak" appearance of the afferent and efferent colonic segments in barium enema. Classic radiographical appearance of "Coffee Bean" sign though apparent in this case, may not be seen in most pediatric cases and is not specific to distinguish from other abdominal pathologies [10].

Management protocol remains a topic of discussion, particularly in a primary emergency setting. In children with no evidence of peritonitis or ischemic bowel, treatment is initiated with resuscitation and detorsion of the sigmoid volvulus, achieved by sigmoidoscopy and rectal tube placement [8]. A barium enema is of therapeutic and diagnostic value [6]. All nonoperative modalities continue to carry a risk of recurrence and perforation [3].

The aim of management is a reduction of the sigmoid volvulus with the prevention of recurrent episodes. The definitive treatment of sigmoid volvulus is sigmoidectomy, either with primary anastomosis or colostomy [3]. Recurrence has been noted to be common when detorsion is done, whereas recurrence after sigmoidectomy has never been reported so far.

If accurate diagnosis and prompt treatment are given to a child presenting with sigmoid volvulus, it continues to have a good prognosis.

## Conclusions

Clinicians and surgeons should watchout and keep a careful eye to avoid missing the diagnosis of sigmoid volvulus by maintaining a high degree of suspicion when a case presents with typical symptoms, irrespective of the patient's age. Delayed management can lead to dire consequences such as gangrene, perforation, and septic shock.

Therefore, it is important to maintain a strong suspicion of sigmoid volvulus even in the pediatric age group presenting with symptoms of acute intestinal obstruction, especially if radiological investigations point toward a diagnosis with the classical sign, as timely management is crucial.
